# Identification of herbal formula Huatanqushihuoxue formula as a potential therapeutic agent for metabolism-related fatty liver disease: a multi-omics and network pharmacology approach

**DOI:** 10.3389/fimmu.2026.1751033

**Published:** 2026-04-02

**Authors:** Sutong Liu, Lihui Zhang, Wanyi Zhu, Weichen Ma, Qing Zhao, Minghao Liu, Wenxia Zhao

**Affiliations:** 1 The First Affiliated Hospital of Henan University of Chinese Medicine, Zhengzhou, China; 2 Collaborative Innovation Center of Prevention and Treatment of Major Diseases by Chinese and Western Medicine, Henan, Zhengzhou, China

**Keywords:** HQHF, MAFLD, molecular docking, network pharmacology, TCM

## Abstract

**Objective:**

This study aimed to evaluate the therapeutic effect of the traditional Chinese medicine compound Huatan Qushi Huoxue prescription (HQHF) on Metabolic Associated Fatty Liver Disease (MAFLD) and explore its underlying mechanisms through a multi-method approach.

**Methods:**

A high-fat diet-induced MAFLD mouse model was established to assess HQHF’s effects on liver pathology, biochemical markers, and oxidative stress. The bioactive components and potential targets of HQHF were identified through network pharmacology. MAFLD-related signaling pathways were explored by integrating data from the Gene Expression Omnibus (GEO) database, followed by molecular docking analyses. For experimental validation, key inflammatory cytokines and proteins (Interleukin-6 (IL-6), Interleukin-1α (IL-1α), Interleukin-1β (IL-1β), Matrix Metallopeptidase 9 (MMP9), Chemokine Ligand 2 (CCL2), and Intercellular Adhesion Molecule 1 (ICAM1)) were measured using Enzyme-Linked Immunosorbent Assay (ELISA), immunohistochemistry (IHC), and immunofluorescence.

**Results:**

Histopathological analysis showed that HQHF significantly alleviated liver steatosis and inflammatory cell infiltration. Biochemical analysis indicated that HQHF reduced serum and liver triglyceride levels, serum total cholesterol, liver enzymes (Aspartate Aminotransferase, AST, and Alanine Aminotransferase, ALT), and malondialdehyde (MDA), while increasing glutathione (GSH) levels. Network pharmacology identified 33 bioactive components and 89 key targets, which were enriched in pathways including Mitogen-Activated Protein Kinase (MAPK), Toll-like Receptor (TLR), Interleukin-17 (IL-17), and Advanced Glycation End product-Receptor for Advanced Glycation End product (AGE-RAGE). Six core genes (MMP9, IL1α, CCL2, IL1β, IL6, ICAM-1) were highlighted. Molecular docking confirmed strong binding of major HQHF compounds (e.g., quercetin, baicalin) to these targets, and experimental assays further validated their downregulation in HQHF-treated mice.

**Conclusion:**

HQHF exerts lipid-lowering and anti-inflammatory effects in MAFLD by regulating multiple targets and pathways related to lipid metabolism and inflammation.

## 1 Introduction

Metabolic dysfunction-associated fatty liver disease (MAFLD) is a leading chronic liver disorder worldwide. It is characterized by excessive fat accumulation in the liver resulting from metabolic dysregulation. The global prevalence of MAFLD is rising alongside obesity and type 2 diabetes mellitus (T2DM). This trend represents a critical public health challenge due to significant metabolic and cardiovascular consequences (1, 2). MAFLD encompasses a spectrum of liver conditions, from simple steatosis to metabolic dysfunction-associated steatohepatitis (MASH). MASH can progress to fibrosis, cirrhosis, and hepatocellular carcinoma, imposing a significant burden on healthcare systems and patient quality of life (3, 4). Despite its high prevalence and severe outcomes, clinical management primarily relies on lifestyle modifications, including diet and exercise. Pharmacological options are limited by poor target specificity and adverse effects, highlighting the urgent need for new, safe, and effective therapies (5).

Recent studies have revealed that insulin resistance, lipotoxicity, oxidative stress, and low-grade chronic inflammation collectively contribute to hepatic injury and fibrogenesis in MAFLD (6, 7). These factors interact via multiple molecular signaling pathways, including MAPK, Toll-like receptors, and IL-17, to exacerbate liver damage and drive disease progression. Despite advances, the intricacies of these molecular signaling networks, particularly under the influence of multi-component therapeutics, remain incompletely understood. Given the complex pathogenesis of MAFLD, therapeutic approaches targeting multiple pathways simultaneously are of great interest. Consequently, Traditional Chinese medicine (TCM) formulations, especially multi-herbal compounds, show promise due to their pleiotropic actions—that is, their ability to affect multiple biological pathways—including anti-inflammatory, antioxidant, and lipid-modulating effects. For example, the multi-ingredient herbal formula HQHF contains bioactive constituents with reported benefits for MAFLD pathogenesis (8). It is composed of 8 natural medicines, each containing multiple natural compounds. HQHF contains high levels of silybin, tanshinone IIA, quercetin, and saikosaponin D, which have liver-protective, anti-inflammatory, anti-oxidation, and anti-lipid metabolism effects (9). So far, clinical and experimental studies have confirmed that HQHF has strong anti-MAFLD activity. HQHF regulates oxidative stress and inflammation in the MASH model and exhibits significant inhibitory effects on MASH through the pyroptosis pathway (10, 11). In addition, clinical trials show that HQHF can reduce ALT levels in MASH patients and improve liver/spleen CT values, thereby achieving the goal of treating MASH (12).

However, its holistic mechanism, involving multi-target and multi-pathway interactions, remains to be systematically elucidated. Network pharmacology has become an important method for elucidating the mechanisms of action of natural medicine and is applicable to the study of multi-target TCM prescriptions. To address this knowledge gap, integrative approaches combining network pharmacology and experimental validation have gained traction in dissecting complex herbal formulations. Network pharmacology systematically identifies active compounds, predicts their potential targets, and highlights relevant biological pathways, and highlights relevant biological pathways to understand the multi-target effects of TCM prescriptions in metabolic diseases (13). Additionally, molecular docking techniques facilitate the assessment of binding affinities between herbal constituents and key protein targets, refining the prediction of therapeutic interactions. These in silico analyses, when coupled with *in vivo* animal models that recapitulate human MAFLD features, offer a robust platform to validate mechanistic hypotheses related to herbal formula mechanisms and therapeutic efficacy (3, 6).

In this study, we used a high-fat diet (HFD)-induced MAFLD mouse model to evaluate the therapeutic potential of HQHF. We focused on its effects on liver histopathology, biochemical parameters, and the inflammatory environment. We complemented this experiment by using comprehensive network pharmacology analyses to systematically screen HQHF’s active components and their molecular targets related to MAFLD pathogenesis. Following these analyses, molecular docking studies were performed to verify the interactions between key herbal compounds and core protein targets predicted to mediate anti-MAFLD effects. To confirm the computational predictions, we used immunohistochemistry and immunofluorescence to assess hepatic expression of key inflammatory and metabolic proteins. This combined methodological approach integrated computational and experimental techniques to clarify the multiple mechanisms through which HQHF acts against MAFLD.

## 2 Materials and methods

### 2.1 Preparation of HQHF

The HQHF formulation was supplied by the Pharmacy Department of the First Affiliated Hospital of Henan University of Chinese Medicine. This formula comprises the following botanical ingredients: 30 grams of Alisma orientalis, 15 grams of Laminaria japonica, 10 grams of Cassia obtusifolia, 15 grams of Curcuma longa, 15 grams of Salvia miltiorrhiza, 15 grams of Crataegus pinnatifida, 6 grams of Bupleurum Chinese, and 15 grams of Silybum marianum. The herbs were immersed in water at a ratio of 1:10 within a multifunctional barrel, followed by heating and filtration to yield the decoction. This decoction was then concentrated to a predetermined volume utilizing a rotary evaporator. The identification of the chemical constituents within HQHF was conducted through Orbitrap high-resolution LC-MS analysis **Supplementary Figure S1**.

### 2.2 Determination of HQHF dosage

In this study, the daily dosage for adults (121 g Herbal Medicine) was converted to an equivalent dosage for mice using a body surface area conversion factor of 9.01, resulting in an equivalent dosage of 18.17 g/kg/d for mice. Taking a mouse weighing approximately 20 g as an example, three intervention dosages were set at low, medium, and high levels, which were 0.18 g/d/mouse, 0.36 g/d/mouse, and 0.72 g/d/mouse, respectively. Our previous extensive basic research has confirmed the efficacy of the three intervention dosages and found that the high dosage had the best efficacy; therefore, this project chose the high dosage of 0.72 g/d/mouse for subsequent experiments.

### 2.3 Animals

The animals used in this study were obtained from Zhejiang Weitong Lihua Experimental Animal Technology Co., Ltd., which meets the required quality standards (SCXK(Zhe)2020-0002). The animal research protocol was approved by the Ethics Committee of Henan University of Chinese Medicine (Animal Ethics Approval No.: IACUC-2023022030), confirming adherence to ethical guidelines. Six-week-old male C57BL/6 mice, weighing 20–25 g, were chosen for the study. The mice were housed in a pathogen-free environment with a 12-hour light/dark cycle, a temperature of 23 ± 2 °C, and humidity of 60 ± 5%, with free access to food and water. The mice were randomly assigned to three groups, each containing 10 mice: a control group, a HFD group, a TCM intervention group, and two additional treatment groups receiving different interventions. Specifically, the HFD and TCM groups were fed a high-fat diet composed of 84% standard feed, 1% cholesterol, 10% lard, and 5% egg yolk powder; this diet was procured from Jiangsu Xietong Biological Technology Co., Ltd. The control group received a standard diet. After 12 weeks of high-fat feeding, the TCM intervention group received the TCM treatment by oral gavage for 4 weeks.

### 2.4 Reagents and consumables

The assay kits for determining the levels of total cholesterol (TC) and triglycerides (TG) in serum, as well as TC, TG, alanine aminotransferase (ALT), aspartate aminotransferase (AST), malondialdehyde (MDA), glutathione (GSH), interleukin-1 alpha (IL1α), interleukin-1 beta (IL1β), and interleukin-6 (IL6) in liver tissues, were procured from Wuhan Elabscience. Antibodies targeting intercellular adhesion molecule 1 (ICAM1), matrix metalloproteinase 9 (MMP9) (GB15132), chemokine ligand 2 (CCL2)(GB112335), IL1β(GB11113), and IL6(GB11117) (all detected in liver tissues) were obtained from Wuhan Saiweier. Additionally, horseradish peroxidase (HRP)-conjugated goat anti-rabbit and goat anti-mouse secondary antibodies were also acquired from Wuhan Saiweier.

### 2.5 Hematoxylin and eosin staining

Frozen tissue sections were removed from storage, thawed, and then fixed before further treatment. HE staining was performed, followed by dehydration and mounting of the sections.

### 2.6 Oil red O staining

Frozen sections were removed from storage, thawed, and then fixed. After rinsing with water, the sections were air-dried. The Oil Red O staining solution was prepared and left to stand overnight before being filtered three times. The sections were then stained. Destaining and washing were performed using isopropanol. Finally, the sections were counterstained with hematoxylin and mounted.

### 2.7 Network pharmacology analysis

The active components of HQHF and their corresponding targets were identified using several databases, including TCMSP (https://tcmsp-e.com/tcmsp.php), SwissTargetPrediction (http://swisstargetprediction.ch), and STITCH (https://string-db.org/). All targets were standardized through the Uniprot database (https://www.uniprot.org) to remove duplicate and irrelevant entries. Additionally, targets associated with MAFLD were obtained from CTD (http://ctdbase.org), DrugBank (https://go.drugbank.com), GeneCards (https://www.genecards.org), and OMIM (https://omim.org). The GSE89632 dataset from the GEO database (https://www.ncbi.nlm.nih.gov/geo) was used to identify differentially expressed genes(DEGs) related to MAFLD. The GSE89632 dataset includes a total of 63 samples, consisting of 20 cases of simple hepatic steatosis, 19 cases of NASH, and 24 cases of healthy living liver. We first used R software (version 4.2.1) and the normalizeBetweenArrays function from the limma package to standardize the data. Based on gene expression profile data, inter-group differential analysis was conducted, with the threshold for screening DEGs set at |LogFC|>0.58 and p.adj<0.05. The R packages and their versions used in this analysis are as follows: GEOquery[2.64.2], limma[3.52.2], ggplot2[3.4.4], ComplexHeatmap[2.13.1].

Subsequently, Cytoscape 3.9.1 software was employed to construct a network diagram illustrating the interactions between drug components and their targets. Network analysis plugins facilitated topological analysis to identify core compounds based on degree values. We analyzed the intersection of drug component targets, disease targets, and DEGs from the GSE89632 dataset. The overlapping targets were considered potential HQHF targets for treating MAFLD and were visualized using a Venn diagram. To elucidate potential molecular mechanisms, Gene Ontology (GO) and Kyoto Encyclopedia of Genes and Genomes (KEGG) pathway enrichment analyses of these core targets were performed using R software (version 4.2.1) with the clusterProfiler package (version 4.4.4), following gene identifier conversion via the org.Hs.eg.db package for Homo sapiens.

### 2.8 Immunohistochemistry

Paraffin-embedded sections were rehydrated in water and then subjected to antigen retrieval procedures. Next, the sections were incubated in a 3% hydrogen peroxide solution at room temperature in the dark for 25 minutes. After rinsing with PBS, the sections were blocked with bovine serum albumin (BSA) and incubated overnight at 4 °C with the primary antibody. Subsequently, they were washed again before applying the secondary antibody, which was incubated at room temperature for 50 minutes. After an additional wash, the 3,3’-Diaminobenzidine(DAB) chromogen was added, and color development was carefully monitored under a bright-field microscope. Hematoxylin counterstaining was performed for about 3 minutes, followed by rinsing with tap water, differentiation with a hematoxylin differentiation solution for a few seconds, and rinsing again with tap water. Finally, the sections were dehydrated, cleared, and mounted. The staining results were then assessed using a bright-field microscope.

### 2.9 Immunofluorescence

Paraffin-embedded sections were deparaffinized and then rehydrated; antigen retrieval was subsequently performed. After cooling, the slides were washed with phosphate-buffered saline (PBS) at pH 7.4. The slides were gently dried, circled with a hydrophobic pen to delimit the staining area, and blocked using BSA prior to the addition of the primary antibody. The sections were incubated overnight at 4 °C in a humidified environment. The following day, a secondary antibody was added and incubated at room temperature in the dark for 50 minutes. After three washes with PBS, 4’,6-diamidino-2-phenylindole(DAPI) staining was performed in the dark at room temperature for 10 minutes. The sections were washed three more times, then treated with fluorescence quenching agent B (FQA-B) for 5 minutes, followed by rinsing under running water for 10 minutes. Finally, the sections were mounted with an anti-fade mounting medium for imaging.

### 2.10 Construction of the protein-protein interaction network

To build the PPI network, we entered common targets related to both the drug and the disease into the STRING database (https://string-db.org/cgi/input.pl). We conducted the analysis using Homo sapiens as the selected organism and applied a protein interaction score threshold of ≥0.70. The resulting PPI network was subsequently imported into Cytoscape version 3.9.0. A comprehensive topological analysis was performed using the Network Analyzer plugin. Additionally, we employed the cytoHubba software to identify hub genes, selecting the top six based on their degree centrality as primary targets. A cluster module analysis using the Molecular Complex Detection (MCODE) plugin was conducted to visually represent the interactions among these identified targets. For molecular docking studies, small molecule structures were acquired from PubChem (https://pubchem.ncbi.nlm.nih.gov/), while protein structures were sourced from UniProt (https://www.uniprot.org/). The protein structures were then prepared using PyMOL. Molecular docking was conducted via CB-Dock (https://cadd.labshare.cn/cb-dock2/php/blinddock.php). Following docking, the final data visualization was carried out using R version 4.2.1, specifically utilizing the R package ggplot2 (version 3.4.4).

### 2.11 Statistical analysis

All statistical analyses in this study were performed using GraphPad Prism 9.5 software, charts were created using R (version 4.2.1), and the puzzle was completed using Adobe Illustrator 2021. Data are presented as mean ± standard deviation (Mean ± SEM). In statistical inference, comparisons among multiple groups first conducted normality and homogeneity of variance tests: when normal distribution is met, one-way ANOVA is used; if variance is homogeneous, Tukey’s HSD test is used for multiple comparisons; if variance is heterogeneous, Games-Howell test is used; when normal distribution is not met, Kruskal-Wallis H test is used, and Dunn’s test is performed for pairwise comparisons. For comparisons between two groups, independent samples t-test (normal and homogeneous variance) or Mann-Whitney U test (non-normal/heterogeneous variance) is selected based on data distribution. All statistical analyses were conducted with a significance level of α=0.05.

## 3 Results

### 3.1 HQHF mitigates dyslipidemia and hepatic lipid accumulation in MAFLD mouse model

To investigate the impact of HQHF on MAFLD, mice were fed a HFD for 12 weeks to establish the MAFLD model, followed by 4 weeks of HQHF treatment. Mice fed a normal diet (NCD) served as the control group.

**Figure 1:**
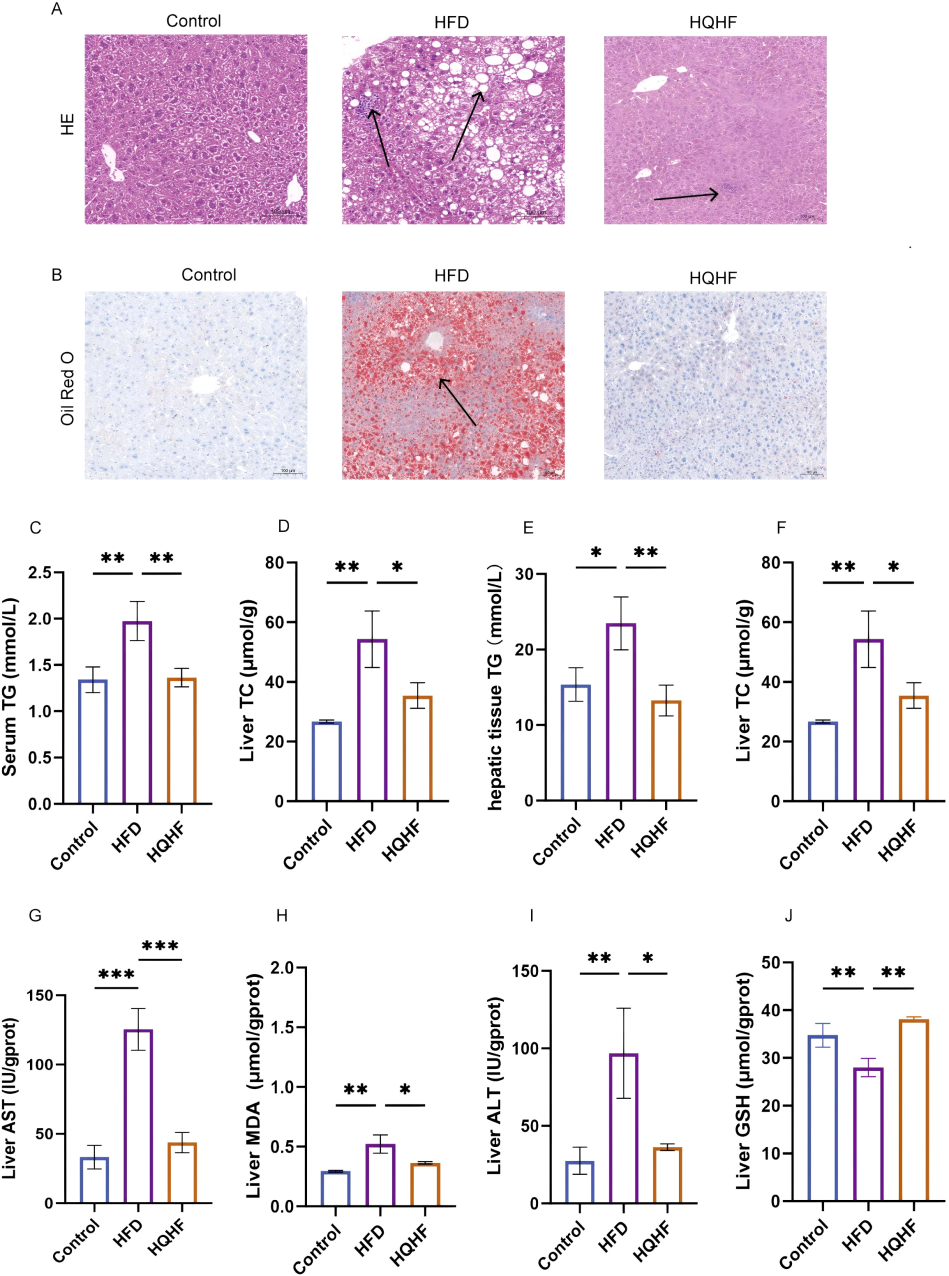
HQHF Mitigates Dyslipidemia and Hepatic Lipid Accumulation in MAFLD Mouse Model **(A)** Representative images of HE staining of liver tissues, Scale bar = 100 µm, 100× magnification. **(B)** Representative images of Oil Red O (ORO) staining, Scale bar = 100 µm, 100× magnification. **(C, D)** Serum levels of TG and TC. **(E–J)** Hepatic biochemical profiles, including TG, TC, serum ALT, AST, MDA, and GSH levels. Data are presented as mean ± SEM. *P < 0.05, **P < 0.01, ***P < 0.001, ns: not significant.

Hepatic inflammation and tissue morphology were examined by HE staining. The results showed that liver tissues from HFD-fed mice had disrupted lobular architecture, with significant inflammatory cell infiltration and lipid vacuoles of various sizes within the cytoplasm. Following HQHF treatment, lipid deposition notably decreased, although some inflammatory cell aggregation remained (**Figure 1A**). Furthermore, Oil Red O staining highlighted the presence of substantial lipid droplets within the liver tissues of HFD-fed mice, while HQHF treatment effectively inhibited the excessive accumulation of these lipid droplets and ameliorated hepatic steatosis (**Figure 1B**). Serum biochemical analyses revealed significantly elevated TG and TC levels in the HFD group, which were reduced by HQHF treatment (**Figures 1C, D**). Biochemical evaluations of liver tissues indicated that HFD-fed mice exhibited increased levels of TG, TC, AST, ALT, and MDA compared to control mice. In contrast, the levels of GSH were diminished in these mice. Treatment with HQHF mitigated HFD-induced hepatic injury, oxidative stress, and lipid accumulation (**Figures 1E–J**). Collectively, these findings suggest that HQHF reduces hepatic lipid deposition and controls oxidative stress in MAFLD mice. Additionally, it reduces liver injury and helps maintain liver enzyme levels.

### 3.2 Network pharmacology analysis of HQHF in MAFLD

**Figure 2:**
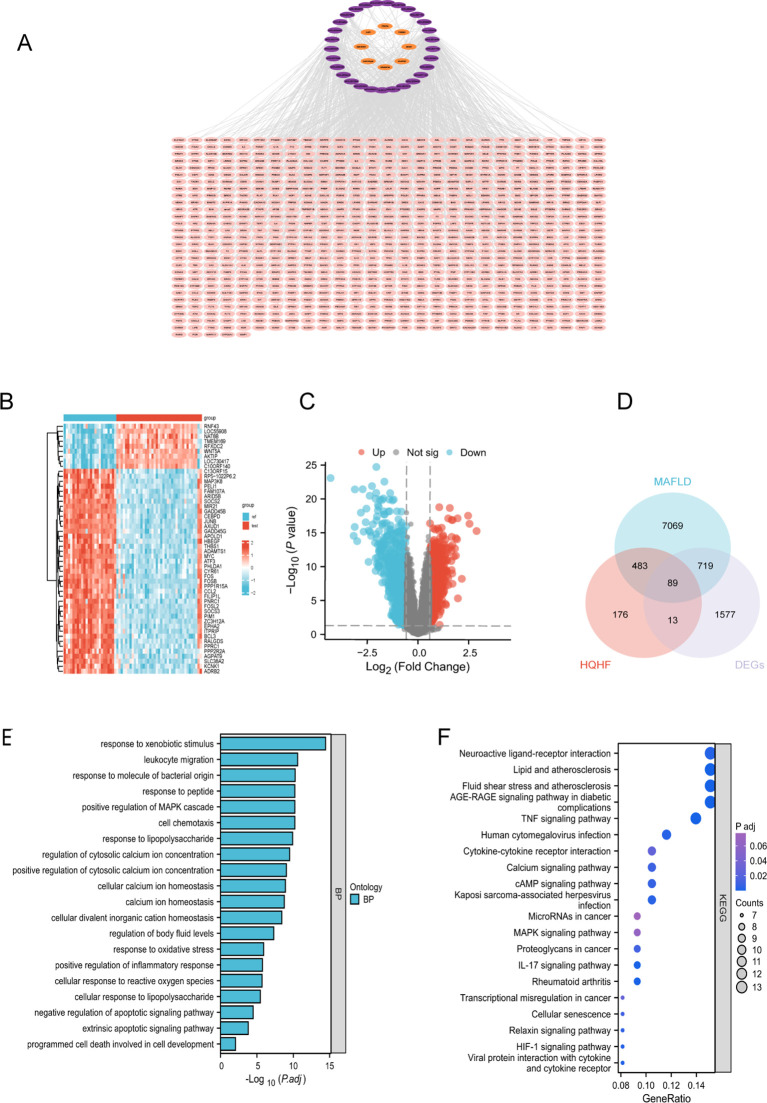
Network Pharmacology Analysis of HQHF in MAFLD. **(A)** Drug-component-target network of HQHF. **(B)** Heatmap of DEGs from dataset GSE89632. **(C)** Volcano plot of DEGs from dataset GSE89632. **(D)** Venn diagram showing the 89 intersecting genes among HQHF targets, MAFLD targets, and DEGs. **(E)** GO enrichment analysis of the 89 intersecting genes. **(F)** KEGG pathway enrichment analysis of the 89 intersecting genes.

To elucidate the underlying mechanisms of HQHF in the context of MAFLD, a comprehensive network pharmacology analysis was performed. The TCMSP, SwissTarget, and STITCH databases together identified 33 active components and 775 unique targets after removing duplicates. The PPI network diagram, illustrating the interactions among HQHF, its active components, and target genes, was constructed using Cytoscape version 3.9.1 (**Figure 2A**). The six active components with the highest degree of connectivity were quercetin, aloe-emodin, baicalin, arachidonic acid, (+)-anomalin, and miltirone.

Furthermore, the CTD database provided 5,200 targets associated with MAFLD. The DrugBank database contributed 13 targets, while the GeneCards and OMIM databases offered 6,146 and 156 potential targets, respectively. After eliminating duplicates, a total of 8,360 potential targets were delineated. The GSE89632 dataset from the GEO database revealed 2,398 differentially expressed genes pertinent to MAFLD (**Figures 2B, C**). By intersecting the targets derived from the herbal components with both the MAFLD-associated targets and the differentially expressed genes from the GSE89632 dataset, 89 common genes were identified (**Figure 2D**). To elucidate the mechanisms by which HQHF mitigates MAFLD, we analyzed 89 identified targets. We performed GO and KEGG enrichment analyses on these targets. The GO analysis highlights significant biological terms such as the positive regulation of the MAPK cascade, responses to oxidative stress, and programmed cell death. These processes are integral to cellular development and underscore the therapeutic potential of HQHF in addressing MAFLD. The KEGG analysis revealed enriched pathways relevant to MAFLD, including AGE-RAGE, lipid metabolism and atherosclerosis pathways, IL-17, MAPK, Toll-like receptor, and non-alcoholic fatty liver disease pathways. Following the enrichment analyses (**Figures 2E, F**).

### 3.3 Validation of hospital of the university of Pennsylvania genes

**Figure 3:**
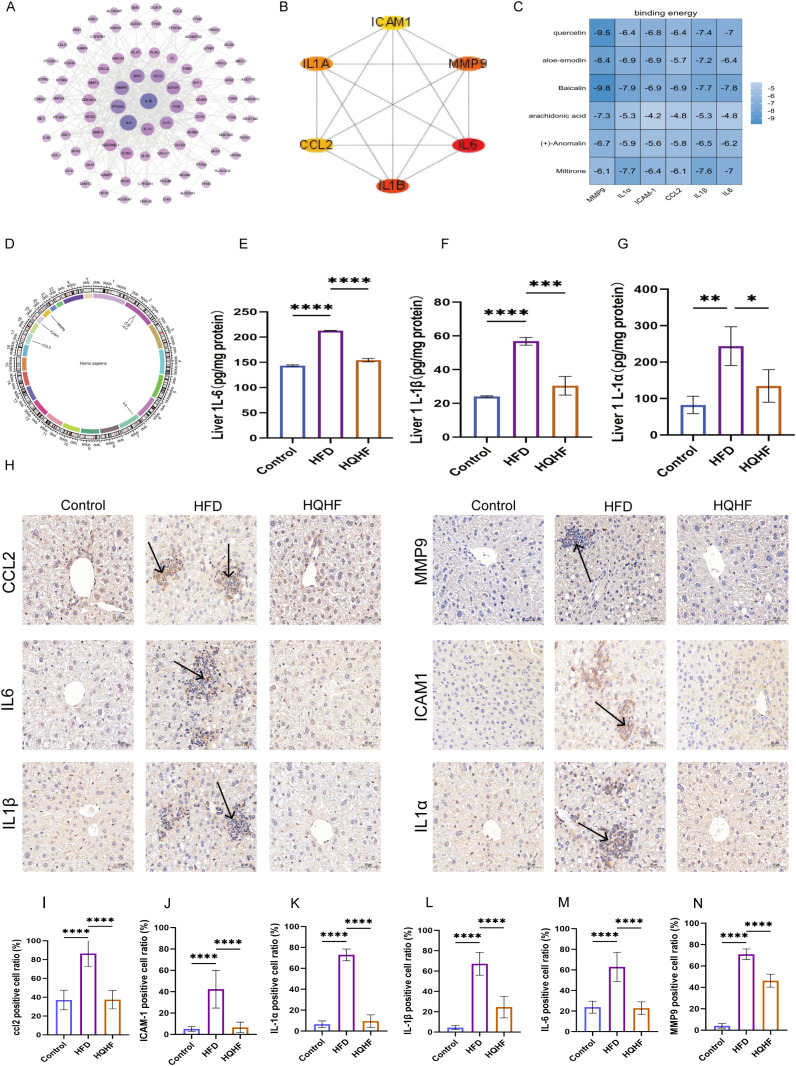
Prediction and validation of the HUP gene. **(A)** PPI network of 89 genes. **(B)** 6 HUP genes. **(C)** Molecular docking results of 6 HUP genes with 6 effective components of HQHF. **(D)** Chromosomal localization of 6 HUP genes. **(E–G)** Cytokines IL-6, IL-1α, IL-1β levels. **(H)** Immunohistochemistry results of HUP genes, Scale bar = 50 µm, 200× magnification. **(I–N)** The quantitative analysis results of immunohistochemistry for the HUP gene. Data are presented as mean ±SEM. ****P < 0.0001.

We further explored the interaction networks among the 89 targets utilizing the STRING database, leading to the construction of a PPI network constructed based on degree values (**Figure 3A**). The top six key hub genes (MMP9, IL1A,CCL2, IL1B, IL6, ICAM1) were identified using the Maximum Clique Centrality (MCC) algorithm, which detects highly interconnected nodes within the protein interaction network, implemented via the Cyto-Hubba plugin (**Figure 3B**). Building on this analysis, we selected six compounds (quercetin, aloe-emodin,baicalin, arachidonic acid, (+)-Anomalin, miltirone) for molecular docking studies with the core targets MMP9, IL1A, CCL2, IL1B,IL6, and ICAM1 (**Figure 3C**). An affinity threshold of < 4.25 kcal/mol indicates binding activity between the ligand and target.Affinities of < 5.0 kcal/mol suggest favorable binding, while affinities of < 7.0 kcal/mol signify robust docking activity. The most favorable docking interactions, measured in kcal/mol, were observed with quercetin-MMP9 (-9.5 kcal/mol), aloe-emodin-MMP9 (-8.4 kcal/mol), and baicalin-MMP9 (-9.8 kcal/mol).

To validate the significant targets identified for MAFLD, we employed immunohistochemical and immunofluorescence techniques. We performed chromosomal localization on the six genes screened from the above research results to analyze their chromosomal positional information (**Figure 3D**). Initially, we measured the levels of IL1A, IL1B, and IL6 in liver tissues utilizing an ELISA kit. The results showed increased concentrations in the MAFLD cohort. Following HQHF intervention, these levels returned to values comparable to those ofthe control group (**Figures 3E–G**). Subsequent immunohistochemical analysis displayed brown staining of MMP9, IL1A, CCL2, IL1B, IL6, and ICAM1 within the model group, particularly in regions marked by pronounced inflammatory cell infiltration. Treatment with HQHF led to a restoration of these levels toward normal levels (**Figure 3H**). We further conducted a quantitative analysis of the immunohistochemistry results, which showed significant overall differences in the positive cell ratios of MMP9, IL1b, IL6, and ICAM1 among the experimental groups. Multiple comparison analysis indicated that compared to the Control group, the positive cell ratios of MMP9, IL1a, CCL2, IL1b, IL6, and ICAM1 in the HFD group were significantly increased. After HQHF treatment, the positive cell ratios of the above indicators in the HQHF group were significantly decreased compared to the HFD group (**Figures 3I–N**).

**Figure 4:**
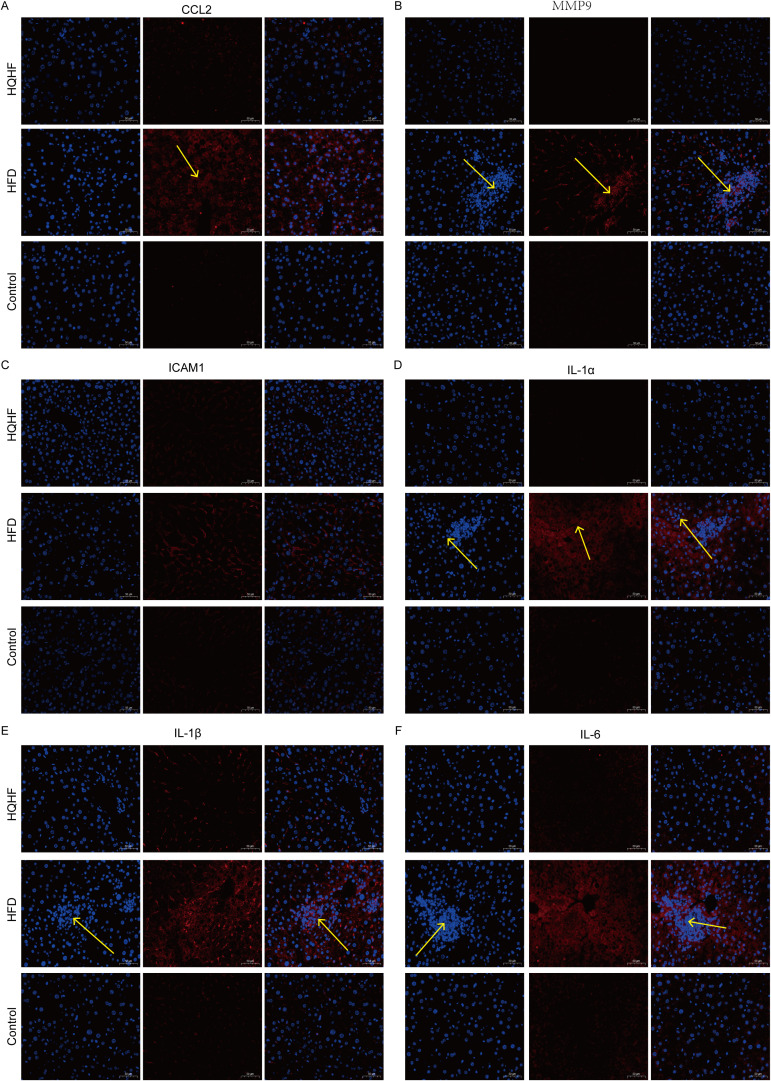
Immunofluorescence results of HUP genes. **(A–F)** CCL2, MMP9, ICAM1, IL1α, IL1β, and IL6 immunofluorescence, Scale bar = 50 µm, 200× magnification.

**Figure 5:**
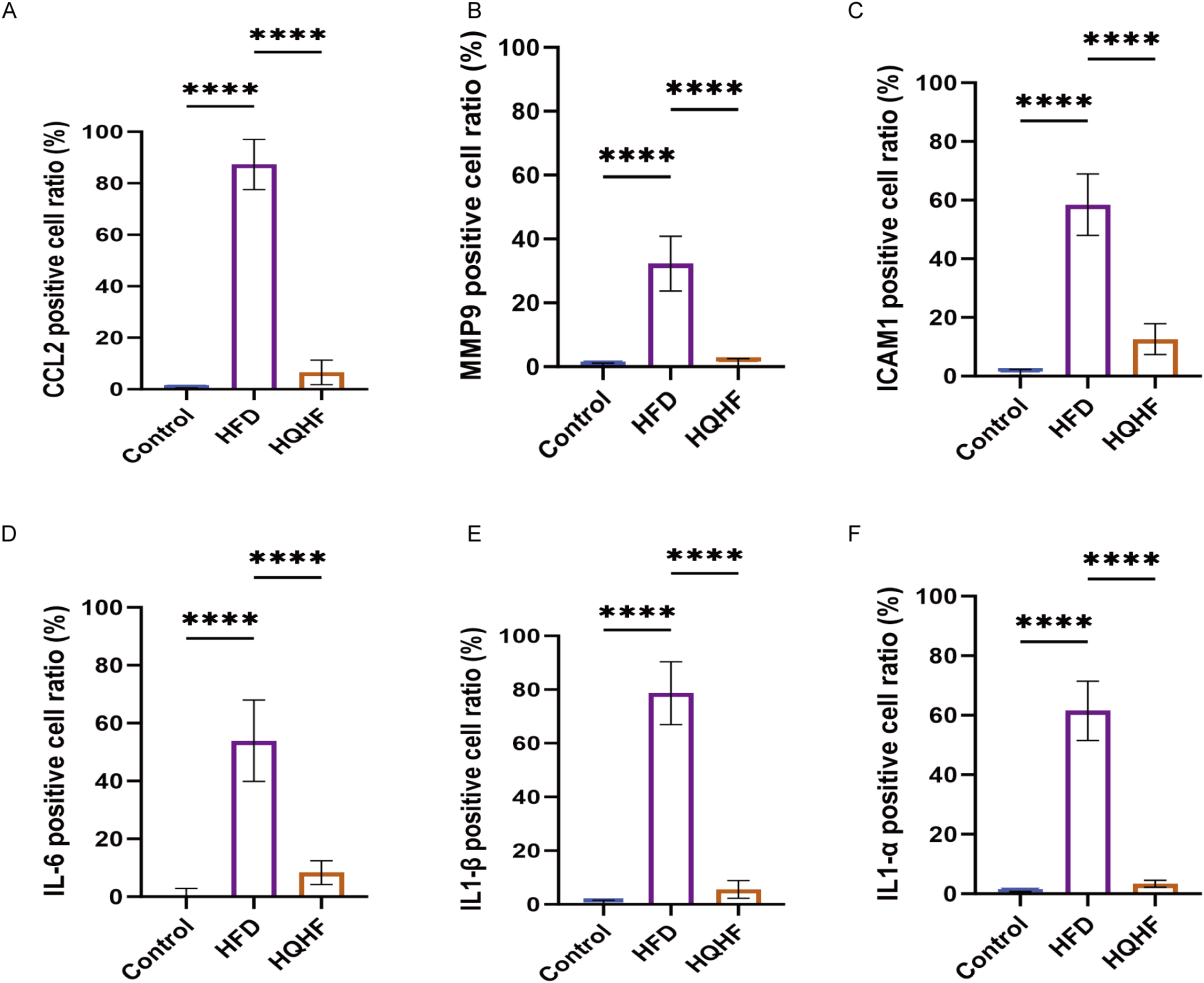
Quantitative analysis of immunofluorescence results of HUP gene. **(A–F)** Quantitative analysis of immunofluorescence results of CCL2, MMP9, ICAM1, IL1α, IL1β, and IL6 Data are presented as mean ±SEM. ****P < 0.0001.

Subsequently, immunofluorescence assessment revealed higher expressions of MMP9, IL1A, CCL2, IL1B, IL6, and ICAM1 in the liver tissue of MAFLD mice. These levels were elevated compared to the control group. Notably, red fluorescence was more pronounced in areas of inflammatory cell accumulation for IL1A, IL1B, and IL6. Treatment with HQHF significantly reduced the red fluorescence expression (**Figures 4A–F**), which was consistent with the IHC results in liver tissue. We further conducted a quantitative analysis of the immunofluorescence results, which showed that compared to the Control group, the positive cell ratios of MMP9, IL1α, CCL2, IL1β, IL6, and ICAM1 in the HFD group were significantly increased. After HQHF treatment, the positive cell ratios of the above indicators were significantly decreased compared to the HFD group (**Figures 5A–F**). We found that the above results were consistent with the immunohistochemistry results of liver tissue.

## 4 Discussion

MAFLD presents considerable health hazards. These include the risk of liver cirrhosis and hepatocellular carcinoma, both of which can profoundly affect patients’ quality of life. Treatment options remain limited, focusing mainly on lifestyle changes and symptomatic relief rather than targeting fundamental pathophysiological mechanisms (14). This situation highlights the urgent need for innovative therapeutic approaches that can effectively target the primary etiological factors of MAFLD. Therefore, our research is particularly relevant and timely as it aims to address these critical challenges.

The histological improvements observed in this study, demonstrated by a significant decrease in hepatic steatosis and inflammatory cell infiltration after HQHF treatment, align with previous research showing that multi-component traditional Chinese medicine formulations can modulate both lipid metabolism and inflammatory responses in MAFLD models (15). Specifically, Oil Red O staining shows a substantial reduction in lipid droplets, and hepatocytes’ structural integrity improves, indicating that HQHF regulates both hepatic steatosis and inflammation. Addressing both metabolic dysregulation and hepatic inflammation is essential for effective management of MAFLD, given the intricate relationship between hepatic steatosis and immune cell activation during disease progression. Moreover, the decrease in inflammatory cell infiltration may reflect a suppression of the pro-inflammatory cytokine milieu, which is associated with the activation of hepatic stellate cells and subsequent fibrogenesis (16). The histological improvements documented in this study reinforce the therapeutic potential of HQHF in mitigating both the initial and progressive pathological features of MAFLD. This offers clear advantages over monotherapy strategies that typically target isolated pathways. Future research needs to clarify whether these histological changes result from modulating specific immune cell populations or enhancing hepatocytes’ lipid handling capacity. Understanding these mechanisms will help explain HQHF’s comprehensive anti-steatotic and anti-inflammatory effects (17).

Integrative analysis of network pharmacology identified 89 pivotal targets that overlap with both active compounds of HQHF and genes associated with MAFLD; these targets are predominantly enriched in signaling pathways including the MAPK signaling pathway, the AGE-RAGE signaling pathway, and Toll-like receptor signaling pathways. These pathways are well-known for regulating oxidative stress, inflammation, and metabolic processes in the liver microenvironment (18–20). Notably, the significantly enriched MAPK cascades play a crucial role in mediating cellular responses to cytokines and oxidative stress (21). Both cytokines and oxidative stress contribute to hepatocyte damage and the activation of hepatic stellate cells. Furthermore, the AGE-RAGE signaling pathway highlights how advanced glycation end-products promote chronic inflammation and fibrosis in MAFLD (22). This pathway links metabolic dysfunction with immunopathology. In addition, the Toll-like receptor signaling pathways emphasize the role of Toll-like receptor signaling in activating innate immunity as a significant factor driving hepatic inflammation (23). Consequently, findings from network pharmacology and bioinformatics provide a molecular basis for the multi-target pharmacological actions of HQHF. These results reinforce the notion that complex herbal formulations are capable of modulating interconnected signaling networks involved in MAFLD pathogenesis. These network pharmacology findings are essential for guiding targeted experimental validation studies and optimizing the composition of HQHF to enhance therapeutic efficacy while minimizing adverse off-target effects (24).

The analysis of the protein-protein interaction network identified six pivotal proteins: MMP9, IL1A, CCL2, IL1B, IL6, and ICAM1. These proteins play a key role in the therapeutic efficacy of HQHF in MAFLD. These proteins serve as essential mediators in hepatic inflammation and extracellular matrix remodeling—processes that fundamentally drive disease progression from steatosis to fibrosis (25–27). The molecular docking studies reveal robust binding affinities between bioactive compounds in HQHF, such as quercetin and baicalin, and these target proteins, offering a plausible mechanistic rationale for the observed anti-inflammatory and antifibrotic properties. For example, the strong interaction between quercetin and MMP9 indicates a potential direct inhibition of MMP9 enzymatic activity; this may reduce matrix degradation and prevent abnormal remodeling. Similarly, interactions with the proinflammatory cytokines IL1B and IL6 may inhibit downstream inflammatory pathways, thus reducing hepatocellular damage and fibrogenesis. These results reinforce the notion of multi-component, multi-target synergy, characteristic of traditional herbal formulations, providing a molecular basis for the systematic refinement of HQHF and its potential translation into clinical applications (28). However, molecular docking is predictive and requires validation by *in vitro* binding assays and functional studies to confirm biological relevance and therapeutic potential.

Experimental validation was performed using ELISA, immunohistochemistry, and immunofluorescence. These methods confirmed the downregulation of key inflammatory mediators and adhesion molecules—such as MMP9, IL1A, CCL2, IL1B, IL6, and ICAM1—in the livers of MAFLD mice treated with HQHF. The agreement between computational predictions and laboratory findings supports the notion that HQHF exerts significant anti-inflammatory effects by targeting key components of the hepatic inflammatory network. Furthermore, spatial distribution analysis using immunofluorescence showed that HQHF specifically reduces inflammation in periportal and perisinusoidal regions, which are important sites for immune cell recruitment and fibrogenesis activation. The reduction in chemokines, such as CCL2, likely contributes to decreased infiltration of monocytes/macrophages, thereby attenuating the escalation of inflammatory responses. Together, these findings highlight the translational importance of HQHF’s multi-targeted anti-inflammatory actions—namely, the simultaneous downregulation of multiple inflammatory mediators and adhesion molecules—and explain its effectiveness in improving liver pathology and function in MAFLD. Future investigations should examine the time course of these protein expression changes and assess downstream signaling modifications to fully characterize the immunomodulatory effects of HQHF.

Biochemical evaluations showed that HQHF treatment markedly ameliorated dyslipidemia in mice with MAFLD. It also reduced the levels of elevated hepatic enzymes and oxidative stress indicators. This was evidenced by decreased serum and hepatic triglycerides, total cholesterol, AST, ALT, and MDA. Additionally, GSH levels were increased. These improvements in metabolic and oxidative parameters highlight HQHF’s ability to restore lipid metabolism and strengthen antioxidant defenses, which breaks the harmful cycle of steatosis and oxidative damage (29). The observed decline in MDA, a byproduct of lipid peroxidation, signified a successful reduction in oxidative damage to cellular membranes (30). The rise in GSH levels pointed to a restoration of cellular redox balance. These findings have important clinical implications, because oxidative stress contributes to hepatocyte apoptosis and fibrogenesis in MAFLD (31). The simultaneous correction of biochemical abnormalities highlights HQHF’s holistic pharmacological profile, which may involve mechanisms targeting lipid metabolism and antioxidant pathways. This profile may offer benefits beyond lipid lowering, such as protection against oxidative and inflammatory damage. Future comparative studies with existing lipid-lowering and antioxidant therapies could clarify HQHF’s relative effectiveness and its potential role in combination drug therapies with other treatments.

In summary, this research outlines the mechanisms by which the TCM formulation HQHF alleviates MAFLD. The study was conducted using a mouse model. These mechanisms involve multiple components, targets, and signaling pathways. The administration of HQHF notably reduced hepatic lipid accumulation, inflammation, oxidative stress, and liver function deficits, while decreasing key pro-inflammatory mediators such as MMP9, IL-1α, IL-1β, and IL6. These results provide strong experimental and theoretical support for HQHF’s efficacy in MAFLD; they highlight its influence on key molecular networks involved in disease progression. The combination of network pharmacology and empirical evidence deepens the understanding of HQHF’s pharmacological effects and provides a valuable framework for developing multi-target therapies for complex metabolic disorders. Building on these findings, future research involving clinical validation, mechanistic studies, and component-specific analyses will be crucial to fully realize HQHF’s clinical potential and optimize its use in metabolic liver diseases.

## 5 Limitations

This study combines *in vivo* experiments, network pharmacology, and molecular docking analysis; however, some limitations warrant cautious interpretation of the results. Relying solely on high-fat diet-induced animal models limits the direct extrapolation of findings to human MAFLD pathophysiology due to interspecies differences and the complexity of human metabolic regulation. The lack of clinical sample validation further restricts the translational relevance of the identified molecular targets and pathways. Additionally, network pharmacology and molecular docking are powerful tools for hypothesis generation. However, their predictions depend heavily on the accuracy and completeness of public databases and computational algorithms. These methods may not fully reflect *in vivo* dynamics and environment-dependent biological interactions. Future research should address these limitations by expanding sample sizes, incorporating clinical cohorts, conducting comprehensive multi-omics validation, and analyzing component interactions. These efforts will be crucial to consolidate insights into the mechanisms of HQHF in MAFLD treatment and enhance its clinical applicability.

## Data Availability

The datasets presented in this study can be found in online repositories. The names of the repository/repositories and accession number(s) can be found below: https://www.ncbi.nlm.nih.gov/geo/, GSE89632.

## Glossary

HQHF: Huatan Qushi Huoxue prescription
MAFLD: Metabolic associated fatty liver disease
GEO: Gene Expression Omnibus
IL-6: Interleukin-6
IL-1α: Interleukin-1α
IL-1β: Interleukin-1β
MMP9: Matrix Metallopeptidase 9
IL1A: Interleukin-1 alpha
CCL2: chemokine ligand 2
IL1B: Interleukin-1 beta
IL6: Interleukin-6
ICAM1: Intercellular Adhesion Molecule 1
ELISA: Enzyme-Linked Immunosorbent Assay
AST: Aspartate Aminotransferase
ALT: Alanine Aminotransferase
MDA: Malondialdehyde
GSH: Glutathione
MAPK: Mitogen-Activated Protein Kinase
IL-17: Interleukin-17
AGE-RAGE: Advanced Glycation End product-Receptor for Advanced Glycation End product
T2DM: type 2 diabetes mellitus
MASH: metabolic dysfunction-associated steatohepatitis
TCM: Traditional Chinese medicine
HFD: high-fat diet
LC-MS: Liquid Chromatography-Mass Spectrometry
TC: total cholesterol
TG: triglycerides
HRP: horseradish peroxidase
HE: Hematoxylin and Eosin
TCMSP: Traditional Chinese Medicine Systems Pharmacology Database and Analysis Platform
CTD: Comparative Toxicogenomics Database
OMIM: Online Mendelian Inheritance in Man
GO: Gene Ontology
KEGG: Kyoto Encyclopedia of Genes and Genomes
PBS: phosphate-buffered saline
IHC: Immunohistochemistry
BSA: bovine serum albumin
DAPI: 4’,6-diamidino-2-phenylindole
FQA-B: fluorescence quenching agent B
DAB: 3,3’-Diaminobenzidine
PPI: protein-protein interaction
MCODE: Molecular Complex Detection
Mean ± SEM: mean ± standard error of the mean
HUP: Hospital of the University of Pennsylvania
MCC: Maximum Clique Centrality.
