# Supplementary figures and images for: Identification of herbal formula Huatanqushihuoxue formula as a potential therapeutic agent for metabolism-related fatty liver disease: a multi-omics and network pharmacology approach
Source: Front Immunol. 2026 Apr 2;17:1751033. doi: 10.3389/fimmu.2026.1751033 (PMC13083021; doi:10.3389/fimmu.2026.1751033)

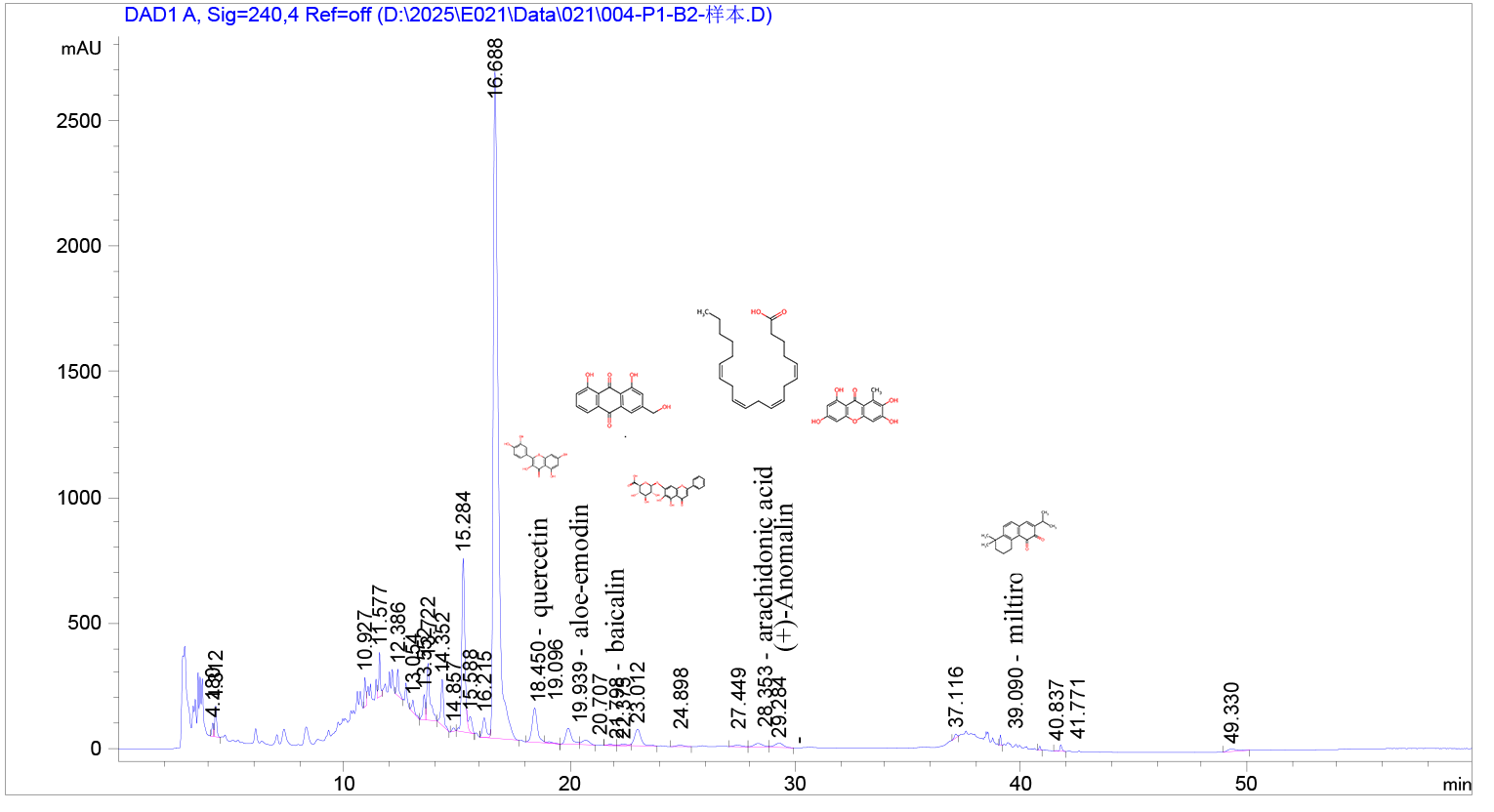

Supplement: Supplementary file 1 [file Image1.tif]
